# Supplementary material for: PRDM16-dependent antigen-presenting cells induce tolerance to gut antigens
Source: Nature. 2025 Apr 14;642(8068):756–65. doi: 10.1038/s41586-025-08982-4 (PMC12176658; doi:10.1038/s41586-025-08982-4)
Supplement: Supplementary file 2 — Reporting Summary [file 41586_2025_8982_MOESM2_ESM.pdf]

nature portfolio

Corresponding author(s): Dan Littman

Last updated by author(s): Mar 31, 2025

Reporting Summary

Nature Portfolio wishes to improve the reproducibility of the work that we publish. This form provides structure for consistency and transparency in reporting. For further information on Nature Portfolio policies, see our Editorial Policies and the Editorial Policy Checklist.

Statistics

For all statistical analyses, confirm that the following items are present in the figure legend, table legend, main text, or Methods section.

n/a	Confirmed	
☐	☒	The exact sample size (*n*) for each experimental group/condition, given as a discrete number and unit of measurement
☐	☒	A statement on whether measurements were taken from distinct samples or whether the same sample was measured repeatedly
☐	☒	The statistical test(s) used AND whether they are one- or two-sided<br>*Only common tests should be described solely by name; describe more complex techniques in the Methods section.*
☒	☐	A description of all covariates tested
☐	☒	A description of any assumptions or corrections, such as tests of normality and adjustment for multiple comparisons
☐	☒	A full description of the statistical parameters including central tendency (e.g. means) or other basic estimates (e.g. regression coefficient) AND variation (e.g. standard deviation) or associated estimates of uncertainty (e.g. confidence intervals)
☐	☒	For null hypothesis testing, the test statistic (e.g. *F*, *t*, *r*) with confidence intervals, effect sizes, degrees of freedom and *P* value noted<br>*Give P values as exact values whenever suitable.*
☒	☐	For Bayesian analysis, information on the choice of priors and Markov chain Monte Carlo settings
☒	☐	For hierarchical and complex designs, identification of the appropriate level for tests and full reporting of outcomes
☒	☐	Estimates of effect sizes (e.g. Cohen's *d*, Pearson's *r*), indicating how they were calculated

Our web collection on statistics for biologists contains articles on many of the points above.

Software and code

Policy information about availability of computer code

Data collection	Flow cytometry was performed on LSR II and Aria using FACSDiva v8.0.1 (BD Biosciences) or a Cytek Aurora using SpectroFLo v3.03 (Cytek Biosciences). Illumina NovaSeq X+ system was used for library sequencing.
Data analysis	BulkRNA-seq were mapped to the murine genome (mm10) with bowtie2 (2.2.3), filtered based on mapping score (MAPQ > 30, Samtools (0.1.19)), and duplicates removed (Picard). Cell Ranger (v7.1) was used to align genomes for single cell experiments. Seurat (v5.1) was used for sc-RNA-seq computational analysis. All code used for analysis in this manuscript is available at https://doi.org/10.5281/zenodo.15032578 and https://doi.org/10.5281/zenodo.15115243.

For manuscripts utilizing custom algorithms or software that are central to the research but not yet described in published literature, software must be made available to editors and reviewers. We strongly encourage code deposition in a community repository (e.g. GitHub). See the Nature Portfolio guidelines for submitting code & software for further information.

Data

Policy information about availability of data

All manuscripts must include a data availability statement. This statement should provide the following information, where applicable:

- Accession codes, unique identifiers, or web links for publicly available datasets
- A description of any restrictions on data availability
- For clinical datasets or third party data, please ensure that the statement adheres to our policy

All mouse and human sequencing data generated and assembled for this project are made available for open-access download at https://doi.org/10.5281/zenodo.15032578 and https://doi.org/10.5281/zenodo.15115243. Reference genomes mm10-2020-A (mouse) and GRCh38-2020-A (human) were used for mapping.

Research involving human participants, their data, or biological material

Policy information about studies with human participants or human data. See also policy information about sex, gender (identity/presentation), and sexual orientation and race, ethnicity and racism.

Reporting on sex and gender	Our limited human biospecimens and public datasets were not designed to test sex or gender, and therefore our findings do not specifically apply to only one sex or gender.
Reporting on race, ethnicity, or other socially relevant groupings	*Please specify the socially constructed or socially relevant categorization variable(s) used in your manuscript and explain why they were used. Please note that such variables should not be used as proxies for other socially constructed/relevant variables (for example, race or ethnicity should not be used as a proxy for socioeconomic status).*<br>*Provide clear definitions of the relevant terms used, how they were provided (by the participants/respondents, the researchers, or third parties), and the method(s) used to classify people into the different categories (e.g. self-report, census or administrative data, social media data, etc.)*<br>*Please provide details about how you controlled for confounding variables in your analyses.*
Population characteristics	Lymph node tissue (our primary human data) was obtained from a de-identified male organ donor at age 22, with no known history of atopy or chronic disease. We utilize public datasets from human intestine (https://doi.org/10.1101/2021.03.28.437379), with male and female donors across ages 63-83 but otherwise de-identified. We utilize public datasets from adult human tonsil (https://doi.org/10.1073/pnas.2318710120) that was otherwise completely de-identified for age and gender.
Recruitment	*Describe how participants were recruited. Outline any potential self-selection bias or other biases that may be present and how these are likely to impact results.*
Ethics oversight	This study does not qualify as human subjects research, as confirmed by NYU Langone Institutional Review Board, because tissues were obtained from a de-identified deceased individual.

Note that full information on the approval of the study protocol must also be provided in the manuscript.

Field-specific reporting

Please select the one below that is the best fit for your research. If you are not sure, read the appropriate sections before making your selection.

☒ Life sciences ☐ Behavioural & social sciences ☐ Ecological, evolutionary & environmental sciences

For a reference copy of the document with all sections, see nature.com/documents/nr-reporting-summary-flat.pdf

Life sciences study design

All studies must disclose on these points even when the disclosure is negative.

Sample size	Three or more mice were used in each experiment. The precise number of animals for each experiment are indicated within each figure legend. These sample sizes were determined from our previous experience in evaluating T cell driven inflammatory responses, and from what is generally accepted in this filed (e.g., PMID 36071167, PMID 36070798).
Data exclusions	No samples were excluded from analysis.
Replication	All the findings on the main figures were replicated at least twice. The precise number of repeats are provided in the figure legend. All attempts were successful.
Randomization	Allocation into sample groups was random. In addition, all control mice were from the same litter. Both males and females were used.
Blinding	Histological analysis was a fully blinded process. However, the remaining experiments were not blinded, since the induction of inflammatory responses versus control experiments, and their serial monitoring, requires re-visiting the same animals in the same cages each day.

Reporting for specific materials, systems and methods

We require information from authors about some types of materials, experimental systems and methods used in many studies. Here, indicate whether each material, system or method listed is relevant to your study. If you are not sure if a list item applies to your research, read the appropriate section before selecting a response.

Materials & experimental systems

n/a	Involved in the study
☐	☒ Antibodies
☒	☐ Eukaryotic cell lines
☒	☐ Palaeontology and archaeology
☐	☒ Animals and other organisms
☒	☐ Clinical data
☒	☐ Dual use research of concern
☒	☐ Plants

Methods

n/a	Involved in the study
☒	☐ ChIP-seq
☐	☒ Flow cytometry
☒	☐ MRI-based neuroimaging

Antibodies

Antibodies used	The following monoclonal antibodies were purchased from Abcam, Thermo Fisher, BD Biosciences or BioLegend: Prdm16 (EPR24315-59), CD3ε (145-2C11), CD4 (RM4-5), CD11b (M1/70), CD11c (N418), CD25 (PC61.5), CD40 (3/23), CD44 (IM7), CD45 (30-F11), CD45.1 (A20), CD45.2 (104), CD62L (MEL-14), CD90.1 (HIS51), CD90.2 (53-2.1), IL-7R (SB/199), CXCR6 (SA051D1), CCR6 (140706), Nkp46 (29A1.4), MHCII I-A/I-E (M5/114.15.2), Ly6G (1A8), Siglec-F (E50-2440), B220 (RA3-6B2), TCR Vα2 (B20.1), TCRβ (H57-597), TCR Vβ5.1/5.2 (MR9-4), TCR Vβ6 (RR4-7), TCRγδ (GL3), Foxp3 (FJK-16s), RORγt (B2D or Q31-378), GATA3 (TWAJ), T-bet (O4-46), BCL6 (K112-91), and IL-22 (IL22JOP). Anti-mouse CD16/32 (Clone 2.4G2, Bio X Cell BE0307R025MG) was used to block Fc receptors. Live/dead fixable blue (ThermoFisher) was used to exclude dead cells. I-Ab OVA328-337 tetramers (HAAHAEINEA) were provided by the NIH Tetramer Core Facility.
Validation	All commercially available antibodies are routinely tested by the vendor.

Animals and other research organisms

Policy information about studies involving animals; ARRIVE guidelines recommended for reporting animal research, and Sex and Gender in Research

Laboratory animals	B6.Cg-Gt(ROSA)26Sortm14(CAG-tdTomato)Hze/J, (Jax 007914), C57BL/6 mice (Jax 000664), CD45.1 mice (B6.SJL-Ptprca Pepcb/BoyJ, Jax 002014), CD90.1 mice (B6.PL-Thy1a/CyJ, Jax 000406) and Cd11ccre mice (B6.Cg-Tg(Itgax-cre)1-1Reiz/J, Jax 008068) were purchased from the Jackson Laboratories. Rorcfl/fl, Rorc(t)gfp/gfp, and Hh7-2tg mice were generated in our laboratory and have been described15,17,43. Il23rgfp mice44 were provided by M. Oukka. OT-II;UBC-GFP mice28,45 were provided by S. R. Schwab. Tg (Δ +3kb Rorc(t)-mCherry), Rorc(t) +6kb-/-and Rorc(t) +7kb-/- mice were generated as described in 'Generation of BAC transgenic reporter and CRISPR knockout mice'.
Wild animals	No wild animals were involved.
Reporting on sex	Both males and females were used in this study. We did not observe any sex-specific phenotypes.
Field-collected samples	There were no field-collected samples.
Ethics oversight	All animal procedures were performed in accordance with protocols approved by the Institutional Animal Care and Usage Committee of New York University School of Medicine.

Note that full information on the approval of the study protocol must also be provided in the manuscript.

Plants

Seed stocks	*Report on the source of all seed stocks or other plant material used. If applicable, state the seed stock centre and catalogue number. If plant specimens were collected from the field, describe the collection location, date and sampling procedures.*
Novel plant genotypes	*Describe the methods by which all novel plant genotypes were produced. This includes those generated by transgenic approaches, gene editing, chemical/radiation-based mutagenesis and hybridization. For transgenic lines, describe the transformation method, the number of independent lines analyzed and the generation upon which experiments were performed. For gene-edited lines, describe the editor used, the endogenous sequence targeted for editing, the targeting guide RNA sequence (if applicable) and how the editor was applied.*
Authentication	*Describe any authentication procedures for each seed stock used or novel genotype generated. Describe any experiments used to assess the effect of a mutation and, where applicable, how potential secondary effects (e.g. second site T-DNA insertions, mosiacism, off-target gene editing) were examined.*

Flow Cytometry

Plots

Confirm that:

☒ The axis labels state the marker and fluorochrome used (e.g. CD4-FITC).

☒ The axis scales are clearly visible. Include numbers along axes only for bottom left plot of group (a 'group' is an analysis of identical markers).

☒ All plots are contour plots with outliers or pseudocolor plots.

☒ A numerical value for number of cells or percentage (with statistics) is provided.

Methodology

Sample preparation	For isolation of cells from lymph nodes and spleens, tissues were mechanically disrupted with the plunger of a 1-ml syringe and passed through 70-µm cell strainers. Bone marrow cells were harvested by flushing out the marrow from cleaned bones using a syringe containing RPMI-1640 wash medium (RPMI-1640 with 3% FBS, 1% GlutaMAX, 1% penicillin–streptomycin, 10 mM HEPES, and 1 mM sodium pyruvate). Red blood cells were lysed with ACK buffer (Thermo Fisher). Cells in bronchoalveolar lavage fluids (BALF) were isolated by flushing the lung with two washes of 0.75 ml PBS via a catheter inserted into a cut made in the trachea.<br>Lung tissues were cut into small pieces and digested in RPMI-1640 wash medium containing 0.5 mg/ml collagenase D (Sigma) and 0.5 mg/ml DNase I (Sigma) at 37°C for 45 min with shaking. After removal of Peyer's patches and cecal patches, the intestines were opened longitudinally, cut into 0.5 cm pieces, and washed in PBS twice. Intestines were then incubated with shaking in HBSS medium (without Ca2+ and Mg2+) containing 3% FBS, 1 mM DTT, 5 mM EDTA, and 10 mM HEPES at 37°C for 20 min twice. After washing with HBSS medium (without Ca2+ and Mg2+) containing 3% FBS and 10 mM HEPES, the tissues were then digested in RPMI-1640 wash medium containing 1 mg/ml collagenase D (Sigma), 0.25 mg/ml DNase I (Sigma), and 0.1 U/ml Dispase (Worthington) at 37°C for 35 min (small intestines) or 55 min (large intestines) with shaking. To isolate leukocytes from the lungs and intestines, the digested tissues were homogenized and passed through 70-µm cell strainers. Mononuclear cells were then collected from the interphase of an 80% and 40% Percoll gradient after a spin at 2,000 rpm for 20 min.
Instrument	Flow cytometric analysis was performed on an LSR II (BD Biosciences) or an Aria II (BD Biosciences).
Software	We used FACSDiva software to collect data, and performed analysis using FlowJo software (Tree Star).
Cell population abundance	Sort purity was determined to be 95% by running post sort sample.
Gating strategy	Naïve Hh7-2tg T cells were sorted as CD4+TCRβ+CD44low/-CD62L+CD25−Vβ6+ (Hh7-2tg).<br><br>Naïve OT-II T cells were sorted as CD4+TCRβ+CD44low/-CD62L+CD25−Vα2+Vβ5.1/5.2+ (OT-II).

☒ Tick this box to confirm that a figure exemplifying the gating strategy is provided in the Supplementary Information.
